# Supplementary material for: A fault-intrusion-tolerant system and deadline-aware algorithm for scheduling scientific workflow in the cloud
Source: PeerJ Comput Sci. 2021 Nov 2;7:e747. doi: 10.7717/peerj-cs.747 (PMC8576554; doi:10.7717/peerj-cs.747)
Supplement: Supplemental Information 6 [file peerj-cs-07-747-s006.zip › Myproject1.1/docs/index-files/index-20.html]

U-Index


JavaScript is disabled on your browser.


- Overview
- Package
- Class
- Use
- Tree
- Deprecated
- Index
- Help

- Prev Letter
- Next Letter

- Frames
- No Frames

- All Classes

A B C D E F G H I J L M N O P Q R S T U V W Z 


## U

UniformDistr - Class in org.cloudbus.cloudsim.distributions
:   A random number generator based on the Uniform distribution.

UniformDistr(double, double) - Constructor for class org.cloudbus.cloudsim.distributions.UniformDistr
:   Creates new uniform distribution.

UniformDistr(double, double, long) - Constructor for class org.cloudbus.cloudsim.distributions.UniformDistr
:   Creates new uniform distribution.

unmapNode(int) - Static method in class org.cloudbus.cloudsim.NetworkTopology
:   Unmaps a previously mapped CloudSim entity to a node in the network topology

updateCloudetProcessingWithoutSchedulingFutureEvents() - Method in class org.cloudbus.cloudsim.power.PowerDatacenter
:   Update cloudet processing without scheduling future events.

updateCloudetProcessingWithoutSchedulingFutureEventsForce() - Method in class org.cloudbus.cloudsim.power.PowerDatacenter
:   Update cloudet processing without scheduling future events.

updateCloudletFinishedSoFar(long) - Method in class org.cloudbus.cloudsim.ResCloudlet
:   A method that updates the length of cloudlet that has been completed.

updateCloudletProcessing() - Method in class org.cloudbus.cloudsim.Datacenter
:   Updates processing of each cloudlet running in this PowerDatacenter.

updateCloudletProcessing() - Method in class org.cloudbus.cloudsim.power.PowerDatacenter
:   Updates processing of each cloudlet running in this PowerDatacenter.

updateCloudletProcessing() - Method in class org.cloudbus.cloudsim.power.PowerDatacenterNonPowerAware
:   Updates processing of each cloudlet running in this PowerDatacenter.

updateCloudletProcessing() - Method in class org.workflowsim.DatacenterExtended


updateDependencies() - Method in class org.workflowsim.clustering.BasicClustering
:   Update the dependency issues between tasks/jobs

updatePeProvisioning() - Method in class org.cloudbus.cloudsim.VmSchedulerTimeShared
:   Update allocation of VMs on PEs.

updateUnderAllocatedMipsForCloudlet(ResCloudlet, double) - Method in class org.cloudbus.cloudsim.CloudletSchedulerDynamicWorkload
:   Update under allocated mips for cloudlet.

updateVmProcessing(double, List<Double>) - Method in class org.cloudbus.cloudsim.CloudletScheduler
:   Updates the processing of cloudlets running under management of this scheduler.

updateVmProcessing(double, List<Double>) - Method in class org.cloudbus.cloudsim.CloudletSchedulerDynamicWorkload
:   Updates the processing of cloudlets running under management of this scheduler.

updateVmProcessing(double, List<Double>) - Method in class org.cloudbus.cloudsim.CloudletSchedulerSpaceShared
:   Updates the processing of cloudlets running under management of this scheduler.

updateVmProcessing(double, List<Double>) - Method in class org.cloudbus.cloudsim.CloudletSchedulerTimeShared
:   Updates the processing of cloudlets running under management of this scheduler.

updateVmProcessing(double, List<Double>) - Method in class org.cloudbus.cloudsim.network.datacenter.NetworkCloudletSpaceSharedScheduler
:   Updates the processing of cloudlets running under management of this scheduler.

updateVmProcessing(double, List<Double>) - Method in class org.cloudbus.cloudsim.power.PowerVm
:   Updates the processing of cloudlets running on this VM.

updateVmProcessing(double, List<Double>) - Method in class org.cloudbus.cloudsim.Vm
:   Updates the processing of cloudlets running on this VM.

updateVmsProcessing(double) - Method in class org.cloudbus.cloudsim.Host
:   Requests updating of processing of cloudlets in the VMs running in this host.

updateVmsProcessing(double) - Method in class org.cloudbus.cloudsim.HostDynamicWorkload


updateVmsProcessing(double) - Method in class org.cloudbus.cloudsim.network.datacenter.NetworkHost
:   Requests updating of processing of cloudlets in the VMs running in this host.

uplinkbandwidth - Variable in class org.cloudbus.cloudsim.network.datacenter.Switch


uplinkswitches - Variable in class org.cloudbus.cloudsim.network.datacenter.Switch


uplinkswitchpktlist - Variable in class org.cloudbus.cloudsim.network.datacenter.Switch


usedPes - Variable in class org.cloudbus.cloudsim.CloudletSchedulerSpaceShared
:   The used PEs.

usedPes - Variable in class org.cloudbus.cloudsim.network.datacenter.NetworkCloudletSpaceSharedScheduler
:   The used PEs.

userId - Variable in class org.cloudbus.cloudsim.network.datacenter.AppCloudlet


UtilizationModel - Interface in org.cloudbus.cloudsim
:   The UtilizationModel interface needs to be implemented in order to provide a fine-grained control
    over resource usage by a Cloudlet.

UtilizationModelFull - Class in org.cloudbus.cloudsim
:   The UtilizationModelFull class is a simple model, according to which a Cloudlet always utilize
    all the available CPU capacity.

UtilizationModelFull() - Constructor for class org.cloudbus.cloudsim.UtilizationModelFull


UtilizationModelNull - Class in org.cloudbus.cloudsim
:   The UtilizationModelNull class is a simple model, according to which a Cloudlet always require
    zero capacity.

UtilizationModelNull() - Constructor for class org.cloudbus.cloudsim.UtilizationModelNull


UtilizationModelPlanetLabInMemory - Class in org.cloudbus.cloudsim
:   The Class UtilizationModelPlanetLab.

UtilizationModelPlanetLabInMemory(String, double) - Constructor for class org.cloudbus.cloudsim.UtilizationModelPlanetLabInMemory
:   Instantiates a new utilization model PlanetLab.

UtilizationModelPlanetLabInMemory(String, double, int) - Constructor for class org.cloudbus.cloudsim.UtilizationModelPlanetLabInMemory
:   Instantiates a new utilization model PlanetLab with variable data samples.

UtilizationModelStochastic - Class in org.cloudbus.cloudsim
:   The UtilizationModelStochastic class implements a model, according to which a Cloudlet generates
    random CPU utilization every time frame.

UtilizationModelStochastic() - Constructor for class org.cloudbus.cloudsim.UtilizationModelStochastic
:   Instantiates a new utilization model stochastic.

UtilizationModelStochastic(long) - Constructor for class org.cloudbus.cloudsim.UtilizationModelStochastic
:   Instantiates a new utilization model stochastic.

A B C D E F G H I J L M N O P Q R S T U V W Z

- Overview
- Package
- Class
- Use
- Tree
- Deprecated
- Index
- Help

- Prev Letter
- Next Letter

- Frames
- No Frames

- All Classes
